# Depression Screening Using Daily Mental-Health Ratings from a Smartphone Application for Breast Cancer Patients

**DOI:** 10.2196/jmir.5598

**Published:** 2016-08-04

**Authors:** Junetae Kim, Sanghee Lim, Yul Ha Min, Yong-Wook Shin, Byungtae Lee, Guiyun Sohn, Kyung Hae Jung, Jae-Ho Lee, Byung Ho Son, Sei Hyun Ahn, Soo-Yong Shin, Jong Won Lee

**Affiliations:** ^1^ College of Business KAIST Seoul Republic Of Korea; ^2^ Carey Business School The Johns Hopkins University Baltimore, MD United States; ^3^ College of Nursing Gachon University Seongnam Republic Of Korea; ^4^ Department of Psychiatry University of Ulsan College of Medicine Asan Medical Center Seoul Republic Of Korea; ^5^ Department of Surgery University of Ulsan College of Medicine Asan Medical Center Seoul Republic Of Korea; ^6^ Department of Oncology University of Ulsan College of Medicine Asan Medical Center Seoul Republic Of Korea; ^7^ Department of Emergency Medicine University of Ulsan College of Medicine Asan Medical Center Seoul Republic Of Korea; ^8^ Department of Biomedical Informatics Asan Medical Center Seoul Republic Of Korea; ^9^ Ubiquitous Health Center Asan Medical Center Seoul Republic Of Korea

**Keywords:** depression, smartphone applications, mental health, breast cancer (neoplasms)

## Abstract

**Background:**

Mobile mental-health trackers are mobile phone apps that gather self-reported mental-health ratings from users. They have received great attention from clinicians as tools to screen for depression in individual patients. While several apps that ask simple questions using face emoticons have been developed, there has been no study examining the validity of their screening performance.

**Objective:**

In this study, we (1) evaluate the potential of a mobile mental-health tracker that uses three daily mental-health ratings (sleep satisfaction, mood, and anxiety) as indicators for depression, (2) discuss three approaches to data processing (ratio, average, and frequency) for generating indicator variables, and (3) examine the impact of adherence on reporting using a mobile mental-health tracker and accuracy in depression screening.

**Methods:**

We analyzed 5792 sets of daily mental-health ratings collected from 78 breast cancer patients over a 48-week period. Using the Patient Health Questionnaire-9 (PHQ-9) as the measure of true depression status, we conducted a random-effect logistic panel regression and receiver operating characteristic (ROC) analysis to evaluate the screening performance of the mobile mental-health tracker. In addition, we classified patients into two subgroups based on their adherence level (higher adherence and lower adherence) using a k-means clustering algorithm and compared the screening accuracy between the two groups.

**Results:**

With the ratio approach, the area under the ROC curve (AUC) is 0.8012, indicating that the performance of depression screening using daily mental-health ratings gathered via mobile mental-health trackers is comparable to the results of PHQ-9 tests. Also, the AUC is significantly higher (*P*=.002) for the higher adherence group (AUC=0.8524) than for the lower adherence group (AUC=0.7234). This result shows that adherence to self-reporting is associated with a higher accuracy of depression screening.

**Conclusions:**

Our results support the potential of a mobile mental-health tracker as a tool for screening for depression in practice. Also, this study provides clinicians with a guideline for generating indicator variables from daily mental-health ratings. Furthermore, our results provide empirical evidence for the critical role of adherence to self-reporting, which represents crucial information for both doctors and patients.

## Introduction

Mental distress can impair treatment processes and outcomes, such as adherence to treatment recommendations, satisfaction with care, and quality of life [1-3]. However, when present, depression is detected less than 30% of the time in cancer patients, mainly due to the time constraints of both patients and clinicians as well as patients’ reluctance to undergo depression screening tests [4,5]. To reduce the burden on patients, researchers have developed simpler screening tools that use only one or two questions, such as the Distress Thermometer and the Patient Health Questionnaire-2 (PHQ-2) [5,6]. However, these screening methods are still problematic when dealing with patients who rarely visit a doctor, because these patients do not have a chance to be tested. To alleviate this problem, doctors recommend that patients track their Patient Reported Outcomes (PROs) on paper as a form of mental-status diary [7,8]. However, the inconvenience of keeping daily mental-health ratings on paper leads to a low rate of using such diaries [7,9].

The rapid increase in the use of mobile phones, and specifically smartphones, has prompted health care providers to consider mobile phone apps as a way to collect mental PROs. Such apps are known as mental-health trackers [10,11]. Despite the potential benefits of mental-health trackers in the setting of oncology treatment, prior studies have focused on evaluating the feasibility of data collection and overall response rates [7,10,12], with only a few studies evaluating the validity of the data in depression screening [11,13]. These validity studies show whether the mood ratings sent via text message can be used as a proxy for depression assessment [13] and whether the scores reported via mobile phones are consistent with the ones reported via a paper-based test using traditional depression screening questionnaires [11]. Therefore, it is still unclear whether the daily mental-health ratings, which are gathered using simple instruments and facial emoticon scales via mobile mental-health trackers, can be used to screen for depression for clinical purposes.

The contribution of our study is mainly threefold. First, we provide a performance evaluation of mobile mental-health trackers. Some researchers have raised concerns about using a simpler and shorter depression screening survey designed for a mobile phone, fearing that it may increase measurement errors. However, we argue that the shorter recall period involved in using a mobile mental-health tracker can compensate for potential measurement errors. Prior studies have shown that the accuracy of human memory substantially decreases as the recall period increases [14-17]. The use of mobile mental-health trackers can reduce the patient recall period since it is possible for patients to easily report mental-health ratings on a daily basis. Our results show that daily mental-health ratings collected via mobile mental-health trackers provide results comparable to those of traditional depression screening tools.

Second, we propose three data-processing approaches (average, ratio, and frequency) for generating indicator variables from daily mental-health ratings and evaluate the performance of screening accuracy among these three approaches. From a practical perspective, there has been no discussion on the approach for transforming daily mental-health ratings reported via mobile phone apps to create indicator variables used for depression screening. Although several studies have conducted analyses using cross-sectional time-series data of patients’ mood ratings, these studies focus on finding factors associated with mood variation [18-20]. Thus, our proposed approaches help clinicians transform daily mental-health ratings reported via a mobile phone app to generate indicator variables for depression screening.

Last, we show the effects of adherence on screening accuracy. The vast amount of daily data collected from patients through mobile mental-health trackers can be a burden for clinicians. Therefore, it is crucial to devise a systematic approach that can automatically distinguish useful data from data that may only increase noise, bias, and variability, which are common pitfalls of mobile data [21].

We note that adherent patients tend to make an extra effort to complete a suggested treatment plan [22], as observed in cancer patients reporting mental PROs in a supportive mental-health care setting. We expect that the patient adherence to self-reporting PROs is positively associated with the quantity and quality of data and thus increases the statistical accuracy of the model [23]. We tested this argument by categorizing patients into higher adherence and lower adherence groups and comparing their screening accuracy.

### Research Setting

**Figure 1:**
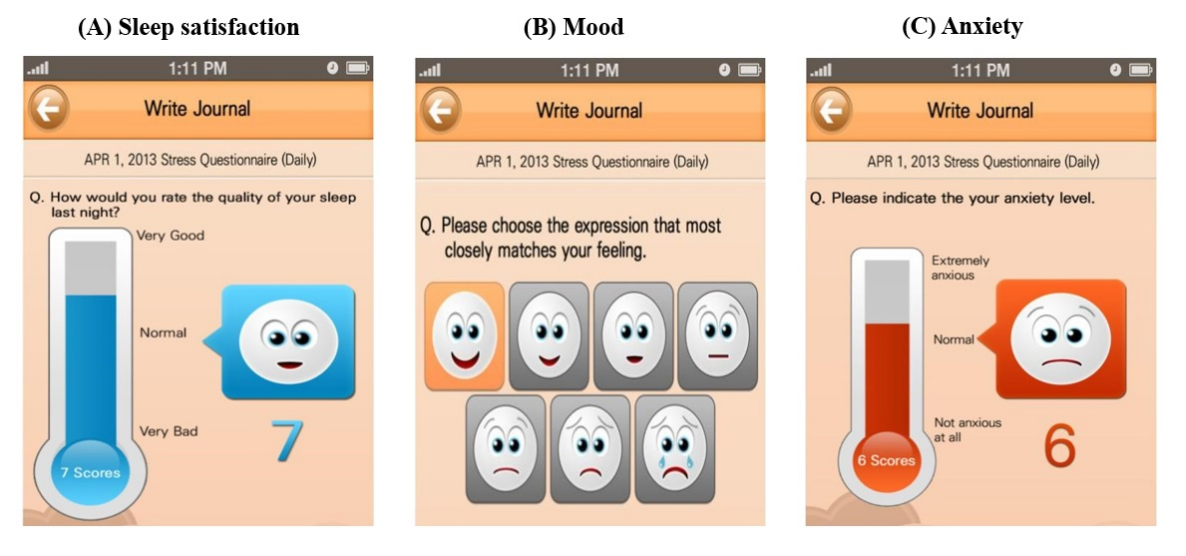
Three mental logs in the Pit-a-Pat app: (A) Sleep satisfaction, (B) Mood, and (C) Anxiety.

In early 2013, the largest hospital in South Korea developed a mobile phone app called Pit-a-Pat [10] (Figure 1), which was designed to collect PROs of breast cancer patients. The patients kept ratings on three mental-health items on a daily basis via the app: anxiety, mood, and sleep satisfaction, which have been shown to be associated with depression [24,25]. These ratings were reported using facial emoticon scales (Figure 1). Patients reported their sleep satisfaction level on a scale ranging from 0 (very bad) to 10 (very good), but this scale was reversed so that it ranged from 0 (very good) to 10 (very bad) in order to make it consistent with other measures, whose values increased with the severity of depression. Patients recorded their mood level on a scale from 0 (none) to 7 (very severe), and anxiety level on a scale from 0 (none) to 10 (very severe).

The app also administered the PHQ-9 test on a biweekly basis. This test is one of the most widely used depression screening tools in primary care settings [24,26-29].

## Methods

Our analysis broadly consists of two parts. One is the evaluation of the accuracy of the mobile mental-health tracker for depression screening. The other is the examination of the effects of adherence on screening accuracy.

### Evaluation of the Empirical Model for Depression Screening

#### Dependent Variable

Depression as a dependent variable was measured based on the PHQ-9 test results. The PHQ-9 test consists of nine items, each scored from 0-3 points, with the final score calculated as the sum of the scores for the nine items. We used a cut-off of 5 points for a depression diagnosis based on prior literature [26,27,29,30]. This relatively low cut-off value reduces the possibility that cancer patients who have depressive tendencies are classified as “normal.” Several studies have reported that depression severity tends to be underestimated in the cancer treatment setting [31-33], despite the high cost of failing to detect depression due to its negative impact on health outcomes [2,3]. For this reason, in the depression treatment setting, researchers put more emphasis on improving a true-positive rate rather than a true-negative rate [34] because it is far more important to correctly identify depressed people than to correctly identify people without depression.

#### Three Data-Processing Approaches for Generating Indicator Variables

We used three approaches to construct the biweekly indicator variables from daily mental-health ratings: (1) average, (2) frequency, and (3) ratio. We generated the indicator variables by making daily mental-health ratings line up with the time interval of PHQ-9 questionnaires, which was biweekly. For instance, using the daily mental-health ratings reported from April 1-14, 2015, we generated the indicator variables and matched them with the PHQ-9 score measured on April 14, 2015, which recorded a patient’s depressive tendencies during the same period.

The average approach calculates the average of each mental-health rating of a patient in a biweekly period. The average approach is easy for doctors to implement because it generates a continuous variable and does not require the doctor to calculate the optimal cut-off values [35]. However, practical guidelines suggest measuring the severity of depression by counting the number of days that people have depressive tendencies during specified periods [24,34].

**Figure 2:**
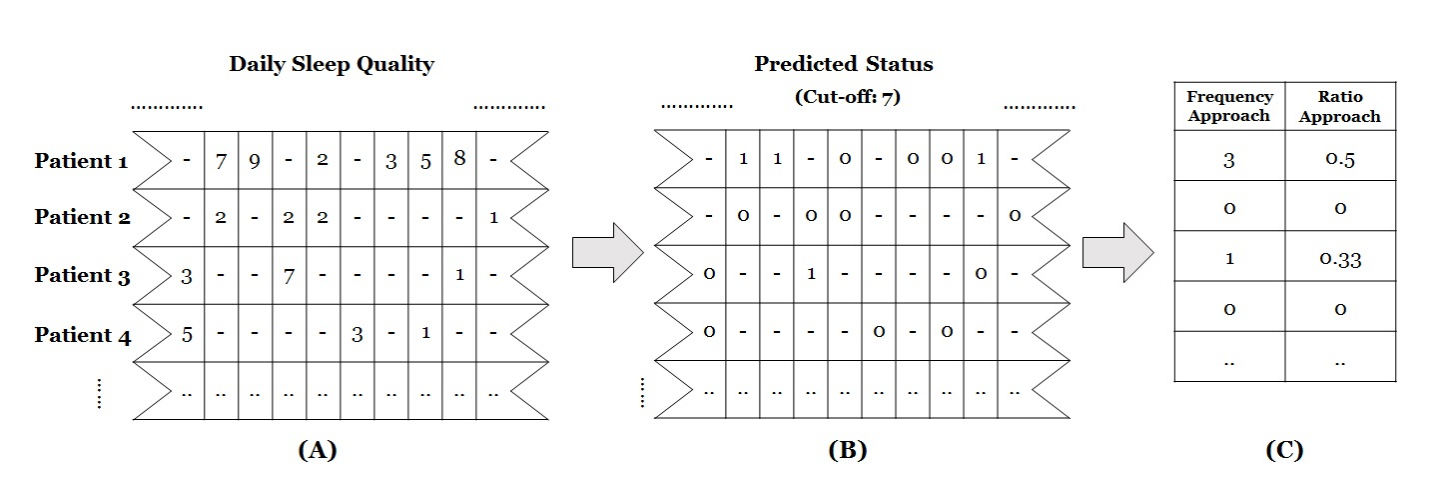
Illustration of data conversion from daily mental-health logs into biweekly indicators with frequency and ratio approaches: (A) Daily scores of sleep quality during 2 weeks, (B) assigned scores of 1 to the days when the reported score is higher than the cut-off value, (C) calculated scores in a biweekly format.

For this reason, we tested two additional approaches: the frequency approach, which counts the number of depressed days during a 2-week period, and the ratio approach, which calculates the ratio of the number of days when the score indicates depression to the total number of days that the ratings are reported during the 2-week period. To construct these indicator variables, we followed two steps to transform the daily mental-health ratings into discrete scales (Figure 2). We first assigned a score of 1 to the days when the reported score on a given day was above a certain cut-off value. For example, if the score for sleep satisfaction on a particular day was higher than the cut-off value (eg, 7 points), we considered the patient to be depressed on that day and assigned the day a value of 1 (Figure 2, Step 2). Thus, each day was assigned a binary value (1=depressed, 0=normal) to indicate whether or not a patient was depressed on that day. Second, the biweekly depressed status was calculated based on each approach (Figure 2, Step 3). For instance, if there are three values of 1 and three values of 0 during a given period (6 days), the indicator variables calculated by the frequency and ratio approaches are 3 and 0.5, respectively. We applied this procedure to all types of daily mental-health ratings. Then, we conducted an analysis on each approach to evaluate the screening performance of the three approaches.

#### Model Specification

Our model is designed to identify depression using indicator variables generated from three types of mental-health ratings:

Depressed_i,t_= Sleep_i,t_+ Mood_i,t_+ Anxiety_i,t_+ *e*_i,t_

where subscripts *i* and *t* indicate each patient and each biweekly period, respectively. The dependent variable, Depressed, takes a binary value (0=normal, 1=depressed). Because we are primarily interested in the extent to which daily mental-health ratings can identify depression, we did not include control variables in the main model. The model parameters were estimated using a random-effect logistic regression model [36-38] (see Multimedia Appendix 1). Logistic regression is one of the classifier models used for estimating the probability of a binary dependent variable based on indicator (ie, independent) variables. We used a random-effect model instead of a fixed-effect model due to the estimation efficiency of the former. Our dataset is a short-panel set, meaning that the number of patients is far greater than the number of time stamps of the observations. Estimation efficiency can be an issue with a fixed-effect model because a fixed-effect model should estimate the parameters of the dummy variables, whose number is the same as the number of patients in our sample. Moreover, our dataset contains some patients who reported a PHQ-9 test result just once during the study period. These patients would be excluded in the analysis of a fixed-effect model. Therefore, a random-effect model is preferred for our situation.

#### Receiver Operating Characteristic Analysis

Receiver operating characteristic (ROC) analysis is used to evaluate the screening accuracy of our model. ROC is a graphic plot, which is widely used to demonstrate the prediction accuracy of a classifier model. It plots the true positive rate (ie, sensitivity) against the false positive rate (ie, 1-specificity) at various threshold values (0< values <1) of predicted probability calculated based on logistic regression models. The area under the ROC curve, referred to as the area under the curve (AUC), represents the probability that a classifier model ranks a positive case higher than a negative case. Therefore, a higher AUC implies a better prediction performance of a classifier model. Rough criteria for assessing the performance of ROCs note that having an AUC higher than 0.7 is considered to be clinically acceptable [39].

#### Procedures for Calculating Cut-Off Points of Each Mental-Health Rating

**Figure 3:**
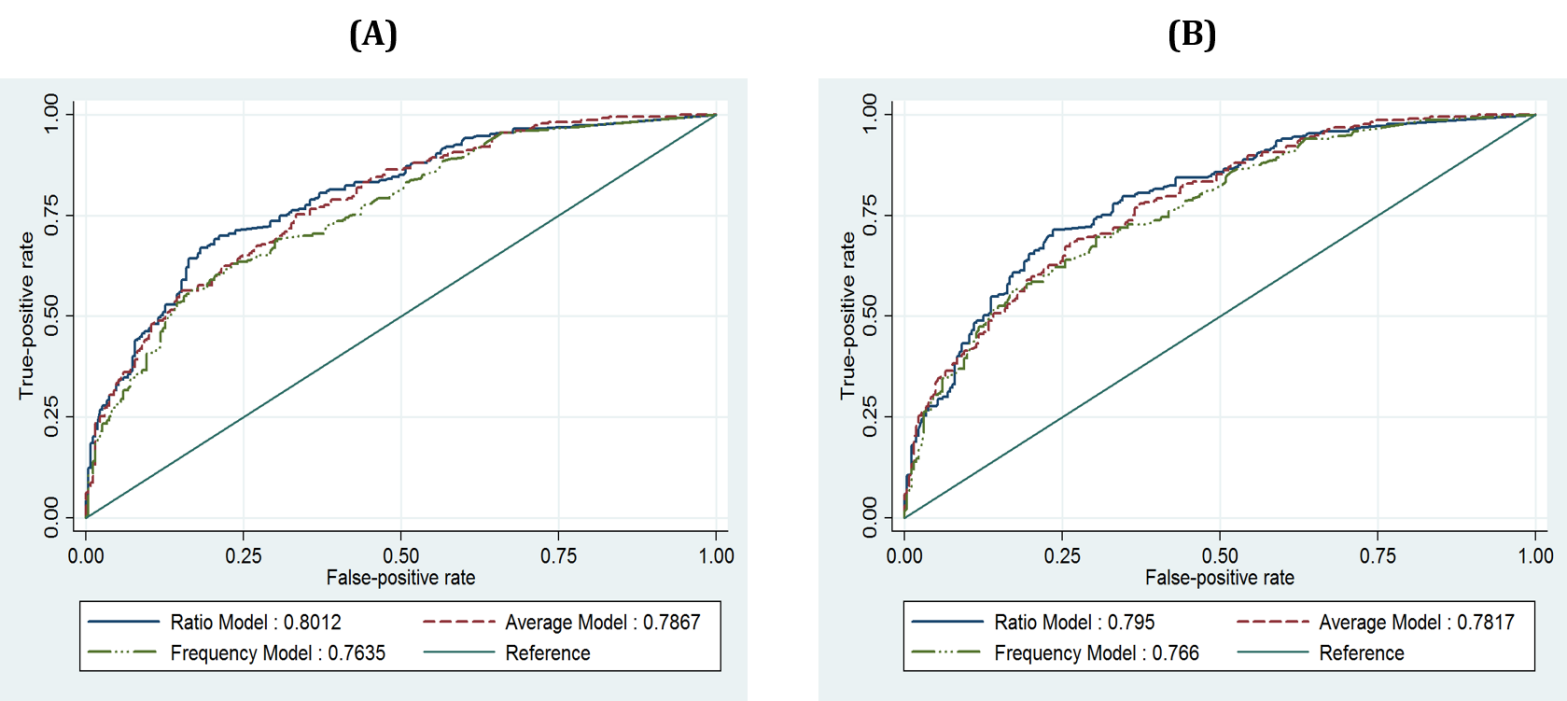
Results of ROC analysis: (A) ROC curves calculated from three models (full samples), (B) ROC curves calculated from three models (subsample excluding the daily logs reported on the day the PHQ-9 is administered).

As discussed earlier, we constructed dichotomy variables for the ratio and the frequency approaches. Dichotomy variables need optimal cut-off values [35] because patients’ mental states should be dichotomized into two groups. We calculated the optimal cut-off value by simulating models. First, we predicted a patient’s mental status by using each daily rating item with an arbitrary cut-off value. Then, we compared the AUCs of all possible cut-off values and selected the one that gives the highest AUC as the optimal cut-off. For example, to determine the cut-off for the Anxiety variable, we (1) selected an arbitrary cut-off value, (2) calculated Anxiety based on the ratio or the frequency approaches (Step 2 in Figure 2), (3) estimated a simplified model, Depressed_it_ = Anxiety_it_ + *e*_it_, and (4) calculated the AUC through ROC analysis. As Anxiety can take a value from 0 to 10, we repeated this procedure 10 times and then selected the cut-off value that produced the highest AUC.

#### Robustness Analyses of Mental-Health Ratings in Detecting Depression

The primary purpose of our empirical analysis is to test the performance of depression screening. Thus, the consistency of the screening accuracy of our model is important. We conducted two additional analyses to ensure the robustness of our results.

First, we conducted a robustness analysis to validate our model by employing the five-fold cross-validation procedure. We (1) randomly partitioned the data into five subsets where the sample size is approximately 100, (2) calculated each cut-off value of indicator variables using four of the subsets as a training set, (3) generated indicator variables for the ratio and frequency approaches, (4) ran a random effect logistic regression using a training set, (5) calculated the predicted probability for the remaining subset as a test dataset, and (6) employed ROC analysis and calculated the AUC. Steps 2-5 were repeated five times by alternating training and test datasets.

Second, one may be concerned with potential bias if patients submitted mental-health ratings on a day they took a PHQ-9 test. Thus, we conducted the analyses using a subsample that excluded the daily ratings reported on the days when a PHQ-9 test was taken.

### Evaluation of Adherence Impact on Screening Accuracy

#### Conceptualization of Adherence to PROs as Composite Construct

In prior studies on adherence to self-reporting in the mobile and Internet health care setting, researchers tended to measure adherence with only one dimension—the response rate to technology during a given period [10,40]. This practice may be too simple because of the multidimensional characteristics of adherence [22,41-44]. We classified the patients into higher and lower adherence groups based on three dimensions: (1) activeness, (2) timeliness, and (3) persistence. Activeness refers to the degree to which the activities of a patient adhere to particular guidelines [22,44]. We calculated Activeness as the total number of days when daily mental-health ratings were reported. For Timeliness, which captures the noncompliance of a patient with treatment plans in the literature [41,42], we counted the total number of days when ratings were reported without delay because the app allowed users to submit ratings for the past few days. Persistence, defined as continuous involvement with clinical treatment during the prescribed period [43], was measured with two variables: (1) the number of biweekly periods between the first and last days in which the patient reported daily ratings (ie, the total duration), and (2) the total number of biweekly periods with reported ratings. The total duration is an important dimension of Persistence because it captures a discontinuity effect if patients stop using the app after a few weeks. However, there could be cases where a patient submits only two ratings, one very early and the other much later in the study period. Therefore, we also considered the total number of biweekly periods with reported ratings. This measure is still different from Activeness because it captures the lower adherence of patients who report ratings very actively during the first few weeks and then subsequently use the app rarely.

We considered a patient’s adherence to using a mobile mental-health tracker as a composite construct of these three factors. These factors (activeness, timeliness, and persistence) address different aspects of adherence, and the relative importance of the three is unclear. Moreover, the way patients adhere to using mobile mental-health trackers can vary depending on their personalities.

#### K-Means Clustering Analysis and Receiver Operating Characteristic Comparison Test

To classify patients based on their adherence levels, we used a *k*-means clustering algorithm (see Multimedia Appendix 1) [45,46]. The *k*-means clustering classifies subjects into homogeneous subgroups, where each observation belongs to the cluster with the smallest intracluster distance and the largest intercluster distance. The number of clusters can be determined based on statistical criteria, such as the Akaike Information Criterion (AIC) [47]. However, a statistical approach often returns so many clusters that it becomes complicated to interpret the characteristics of clusters. For this reason, a researcher’s judgment is also often used [46]. We classified patients into two clusters (ie, higher- and lower-adherent patients) for easier interpretation of results. We also examined the results with three clusters as a robustness check, as we describe below.

After patients were classified into higher- and lower-adherence groups, we compared the AUCs of each group in an ROC analysis. In addition, to support our approach to measure adherence as a multidimensional variable, we compared the AUCs of high- and low-adherence groups when the groups are classified based on prior studies [10,40], using the response rate only when the groups are classified based on our approach using activeness, timeliness, and persistence.

#### Robustness Analyses of Impact of Adherence on Screening Accuracy

To test the robustness of our finding that the screening accuracy is higher for patients with a higher level of adherence, we examined two potential sources of bias that may affect our ROC comparison tests—the length of data collection periods by patients and the number of clusters.

First, we examined whether the difference in the length of data collection periods by patients influences the results. Because each patient started using the app at a different time during the study period, the measure of persistence can be biased for patients who started using the app very early or very late in the study period. For example, persistence can be underestimated for patients who started using the app later in the study period. Likewise, persistence can be overestimated for patients who started using it earlier. Therefore, we examined whether our results are robust if we consider only the rating data collected during the first 24-week period (ie, half of the total study period) for each patient. We also analyzed the subsample excluding patients who joined the study during the last 12 weeks, which was the average usage period of the patients in our sample.

Second, we examined whether the results are maintained when patients are classified into three groups instead of two. This analysis also allows us to address the possibility of outliers in each group (high and low) who may have skewed the results.

## Results

### Sample Description

A total of 85 breast cancer patients provided informed consent to participate in this study (Institutional Review Board No. 2012-0709). These patients submitted 5817 daily mental-health ratings from early April 2013 to late March 2014. We excluded 25 ratings reported by 7 patients who did not complete a PHQ-9 test. As a result, 5792 daily mental-health ratings reported by 78 patients during 24 biweekly periods were used for our analysis. The 78 patients in our sample provided 497 PHQ-9 test results, which consisted of 270 normal statuses and 227 depressed statuses when using a cut-off score of 5 [26,27,29,30]. On average, there were 6.4 observations per patient, with the number of observations ranging from 1 (n=11) to 24 (n=1). The cumulative percentage of the number of days in which patients report ratings at least 11 days during 2 weeks (14 days) is 65.59%. Table 1 shows the demographic information for the 78 patients in our sample.

**Table 1:** Participant characteristics in the two study groups.

Characteristic		Total, n (%) or mean (SD) (n=78)		Lower adherence, n (n=58)		Higher adherence, n (n=20)		*P* ^a^	
**Age, years**									
	Mean (SD)	44.35 (7.01)		44.24 (7.07)		44.65 (7.02)		.83 (*t*)
	≤39	18 (23.1%)		14		4			
	40-49	40 (51.3%)		28		12		.66 (χ²)	
	≥50	20 (25.6%)		16		4			
**Cohabitation** ^b^									
	No	17 (21.8%)		11		6		.31 (χ²)	
	Yes	61 (78.2%)		47		14			
**Children**									
	None	12 (15.4%)		10		2			
	1	17 (21.8%)		12		5		.45 (χ²)	
	2	43 (55.1%)		33		10			
	3 or 4	6 (7.7%)		3		3			
**Marital status**									
	Divorced	3 (3.8%)		3		0			
	Single	6 (7.7%)		5		1		.49 (χ²)	
	Married	69 (88.5%)		50		19			
**Education level**									
	Up to high school	37 (47.4%)		29		8		.44 (χ²)	
	College degree or higher	41 (52.6%)		29		12			
**Employed**									
	Yes	46 (59.0%)		32		14		.25 (χ²)	
	No	32 (41.0%)		26		6			
**Adherence dimension** ^c^									
	Activeness	68.55 (60.06)		37.66		158.15		<.001 (*F*)	
	Timeliness	51.31 (44.08)		29.81		113.65		<.001 (*F*)	
	Duration	6.45 (5.67)		3.79		14.7		<.001 (*F*)	
	Persistence	6.58 (5.76)		3.67		14.5		<.001 (*F*)	

^a^Tested null hypotheses: *t* test: lower and higher adherence groups have the same mean; χ² test: characteristic categories and adherence groups are independent; *F* test: lower and higher adherence groups have the same mean.

^b^Cohabitation refers to patients living with family members.

^c^Key variables by groups are classified using *k*-means clustering (see Results section).

Table 2 shows the summary statistics of the daily mental-health ratings and indicator variables, which are obtained based on the ratio approach. Also, the calculated optimal cut-off values for each indicator variable of the ratio and the frequency approaches are listed in Table 2. For both the ratio and frequency approaches, the cut-off scores for sleep, anxiety, and mood were identified as 7, 6, and 4 points, respectively. Table 3 shows the correlation matrix of these variables.

**Table 2:** Summary statistics of daily mental-health ratings and indicator variables based on the ratio approach.

	n	Mean	SD	Min.	Med.	Max.	Skew.	Kurt.	Cut-off^d^
Sleep rating^a,b^	5792	4.99	2.03	1.00	5	10	–0.10	2.685	‒
Mood rating^a^	5792	3.19	1.29	1.00	3	7	0.372	2.832	‒
Anxiety rating^a^	5792	4.21	2.07	0.00	5	10	–0.075	2.295	‒
Depressed	497	0.46	0.50	0.00	0.00	1.00	0.174	1.030	5
Sleep^b,c^	497	0.21	0.23	0.00	0.14	1.00	1.167	3.951	7
Mood^c^	497	0.40	0.38	0.00	0.33	1.00	0.388	1.590	4
Anxiety^c^	497	0.30	0.32	0.00	0.2	1.00	0.801	2.474	6

^a^Daily mental-health ratings.

^b^Sleep rating and Sleep indicate sleep dissatisfaction (the reversed scale of sleep satisfaction).

^c^The indicator variables based on the ratio approach.

^d^The cut-off value of Depressed is selected based on prior literature. The cut-off values for Sleep, Mood, and Anxiety are calculated based on the simulation analysis described in the Methods section. The cut-off values obtained by using the frequency and average approaches are the same.

**Table 3:** Correlation matrix of daily mental-health ratings and indicator variables by the ratio approach.

	Depressed	Sleep rating	Mood rating	Anxiety rating
Sleep rating^a,b^	‒	1	‒	‒
Mood rating^a^	‒	0.62	1	‒
Anxiety rating^a^	‒	0.48	0.61	1
	Depressed	Sleep	Mood	Anxiety
Depressed	1.00	‒	‒	‒
Sleep^b,c^	0.36	1.00	‒	‒
Mood^c^	0.42	0.38	1.00	‒
Anxiety^c^	0.40	0.22	0.47	1.00

^a^Correlation matrix of daily mental-health ratings.

^b^Correlation matrix of indicator variables by the ratio approach.

^c^Sleep rating and Sleep indicate sleep dissatisfaction (the reversed scale of sleep satisfaction).

### Evaluation of the Empirical Model for Depression Screening

#### Performance of Each Approach to Data Processing in Detecting Depression

Table 4 presents the results of our model with three different approaches to constructing indicator variables. With the ratio approach, all three types of mental-health ratings are statistically significant (*P* ≤.001) in predicting the mental status of patients. This result indicates that each type of mental-health rating addresses different dimensions of patient mental status. For example, consider the case in which two patients reported the same level of anxiety and mood condition but a differing sleep condition. Our result suggests that holding other variables fixed, a one-tenth-unit (0.1) increase in Sleep (ie, an increase in the ratio of depressed days to the total number of reported days in a given biweekly period by 0.1) is associated with a 31.3% increase in the likelihood of the patient being depressed, since exp(0.272)=1.313. Similarly, all other things being equal, a one-tenth-unit increase in Mood and Anxiety is associated with about a 19% increase in the likelihood of the patient being depressed. Likewise, all three types of mental-health ratings with the average approach are statistically significant (*P*<.05), and a one-tenth-unit increase in Sleep *,* Mood *,* and Anxiety increases the odds of the patient being depressed by 4%, 7%, and 4%, respectively. The Sleep and Mood ratings with the frequency approach are statistically significant (*P*<.05). However, the Anxiety rating is not significant. A one-tenth-unit increase in Sleep *,* Mood, and Anxiety in the frequency model is related to increases of about 1%, 2%, and 1%, respectively, in the likelihood of the patient being depressed.

**Table 4:** Results of random effect logistic panel regression^a^(the 497 observations were constructed from the 5792 daily mental-health ratings reported via the mental-health tracker).

	Ratio (*P*)	Average (*P*)	Frequency (*P*)
Sleep	2.722 (<.001)	0.348 (.036)	0.139 (.046)
Mood	1.783 (.001)	0.728 (.004)	0.177 (.001)
Anxiety	1.782 (.001)	0.396 (.005)	0.080 (.133)
Constant	–1.965 (<.001)	–6.002 (<.001)	–1.404 (<.001)
Observations, n	497	497	497
Patients, n	78	78	78

^a^Dependent variable: Mental status, which is 0 if normal (PHQ-9 score <5) and 1 if depressed (PHQ-9 score ≥5).

Chart A in Figure 3 shows the ROC curves of the predicted results of three models and the corresponding AUCs. The AUCs calculated from the ROCs of the ratio, the average, and the frequency approaches are 0.8012, 0.7867, and 0.7635, respectively. The AUC of the ratio approach is not statistically different (*P*=.150) from that of the average approach, but it differs significantly (*P*=.001) from that of the frequency approach. This result shows that the accuracy of depression screening by using the ratio and the average approaches is statistically indifferent, while the accuracy by using the frequency approach is slightly lower in our empirical result.

#### Robustness Analyses of Mental-Health Ratings in Detecting Depression

First, we conducted a five-fold cross-validation test. With the ratio approach, the AUCs of the five subsets range from 0.7228 to 0.8568. The aggregated result of five subsets yields an AUC of 0.7836. With the average approach, the AUCs range from 0.7234 to 0.8488, and the AUC of the aggregated result is 0.7755. The AUCs of the frequency approach range from 0.7107 to 0.8188, and the AUC of the aggregated result is 0.7385. These results suggest that the risk of overfitting is low for our model according to rough criteria that AUCs higher than 0.7 are clinically acceptable [39].

Second, we conducted the analyses using a subsample excluding the daily ratings reported on the day the PHQ-9 is administered. The subsample consists of 480 observations taken from 5022 daily ratings, which still leaves a sufficient number of daily ratings for our analysis. All coefficients of the new analysis results using the subsample with the three approaches are statistically significant, with similar magnitude (*P*<.05), thus confirming the results of the main analysis. Chart B in Figure 3 shows the ROC curves and corresponding AUCs. The resulting AUC is 0.795 with the ratio approach, 0.7817 with the average approach, and 0.766 with the frequency approach. The difference between the AUCs of the ratio and of the average approach is not statistically significant (*P*=.197), but the one between the ratio and the frequency approach is statistically significant (*P*=.02). This result is consistent with the main analysis, indicating that the accuracy of depression screening of all three approaches is acceptable (AUC >0.7), while the results using the ratio and the average approaches are statistically higher than that of the frequency approach.

### Evaluation of Adherence Impact on Screening Accuracy

#### Effect of Adherence to Self-Reported PROs on Screening Accuracy Based on Composite Construct

Table 1 provides descriptive statistics of four variables (ie, Activeness, Timeliness, Duration, and Persistence) used to determine a patient’s adherence level. Among the 78 study patients, 58 and 20 were classified into the lower and higher adherence groups, respectively. The 497 observations in the biweekly panel dataset comprised 208 observations in the lower adherence group and 289 observations in the higher adherence group. The analysis of variance (ANOVA) test results show that the differences in the means of the four variables between the two are statistically significant (*P*<.001). Also, we conducted a *t* test and a Pearson’s chi-square test to examine whether the classification result was associated with other latent factors (see Table 1), such as a patient’s baseline severity of depression and demographics. The results show that the demographic variables are not significantly related to the adherence level (*P*>.05).

**Figure 4:**
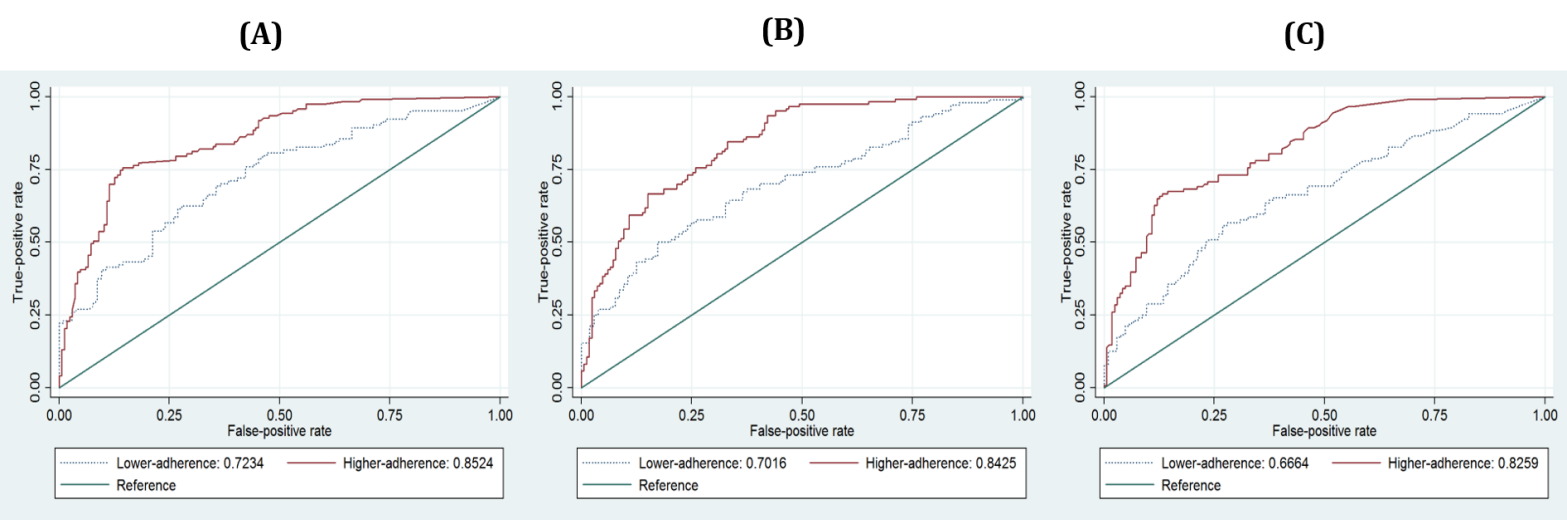
Graphs for ROC comparisons of subsamples by adherence level: (A) ROCs by adherence levels with the ratio approach, (B) ROCs by adherence levels with the average approach, (C) ROCs by adherence levels with the frequency model.

Figure 4 and Table 5 (see Composite construct) show the results of the ROC comparison test by adherence level measured according to our suggestion. These results show the AUCs calculated by the ratio (higher: 0.8524, lower: 0.7234), the average (higher: 0.8425, lower: 0.7016), and the frequency (higher: 0.8529, lower: 0.6664) approaches, respectively. All AUCs of the higher adherence group are statistically higher (*P*<.01) than those of the lower adherence group. These results support our argument that adherence to self-reporting is associated with an increase in the accuracy of depression screening.

**Table 5:** Result for ROC comparisons of subsamples by adherence level (null hypothesis: χ²test, AUCs of higher and lower adherence groups are the same).

Layers	Adherence (n=number of observations)	Ratio approach		Average approach		Frequency approach	
		AUC	*P*	AUC	*P*	AUC	*P*
Composite construct^a^	Lower (n=208)	0.7234	.002	0.7016	.001	0.6664	<.001
	Higher (n=289)	0.8524		0.8425		0.8259	
Prior method^b^	Lower (n=138)	0.7594	.269	0.7290	.104	0.6588	.002
	Higher (n=359)	0.8113		0.8076		0.8198	

^a^Adherence is clustered based on composite constructs of three factors: Activeness, timeliness, and persistence.

^b^Adherence is clustered based on the response rate during 2 weeks.

#### Effect of Adherence to Self-Reported PROs on Screening Accuracy Based on Prior Method

Table 5 (see Prior Method section) presents the result of the comparison of the AUCs when the adherence level is measured based on the response rate during a 2-week period (ie, the number of days when ratings are reported, to 14 days) following prior studies [10,40]. When we use the response rate only, the results show the AUCs calculated by the ratio (higher: 0.8113, lower: 0.7594), the average (higher: 0.8076, lower: 0.7290), and the frequency (higher: 0.8198, lower: 0.6588) approaches. While the comparison of AUCs with the frequency approach is statistically different between high- and low-adherence groups (*P*<.01), those with the ratio and the average approaches are not significantly different (*P*>0.1). These results show that adherence measured based on only one dimension, the response rate, is not sufficient to distinguish two groups that produce different qualities of PROs in terms of screening accuracy. On the other hand, our approach to measure adherence using three dimensions classifies patients into two distinct groups, supporting our suggestion.

#### Robustness Analyses of Impact of Adherence on Screening Accuracy

First, we examined whether our results are robust if we consider only the PROs collected during the first 24-week period (ie, half of the total study period) for each patient (see Table 6). This subsample analysis shows that screening accuracy with all three approaches is statistically higher (*P*<.05) for patients in the higher adherence group than for the ones in the lower adherence group. The analysis results with the subsample excluding patients who joined the study during the last 12 weeks are also consistent with our main results (*P*<.05).

Second, we examined whether our results are maintained when patients are clustered into three groups. The ANOVA test results support that the differences between three groups are statistically significant (*P*<.001). These results show that the screening accuracy is higher for patients with a higher level of adherence (*P*<.05) (see Table 6).

**Table 6:** Results obtained in the robustness analysis.

Layers	Adherence (n=number of observations)	Ratio approach		Average approach		Frequency approach	
		AUC	*P*	AUC	*P*	AUC	*P*
6 months^a^	Lower (n=161)	0.728	.016	0.7239	.033	0.6774	.007
	Higher (n=336)	0.8364		0.8205		0.8088	
Without late starters^a^	Lower (n=171)	0.7405	.006	0.7015	.001	0.6796	.002
	Higher (n=273)	0.8599		0.8540		0.8283	
3 groups^b^	Lower (n=113)	0.6767	.006	0.7134	.020	0.6003	.002
	Middle (n=159)	0.7893		0.7542		0.7986	
	Higher (n=225)	0.8512		0.8446		0.8114	

^a^Null hypothesis: χ²test. The AUCs of higher and lower adherence groups are the same.

^b^Null hypothesis: χ²test. The AUCs of higher, middle, and lower adherence groups are the same.

## Discussion

### Principal Findings

This study provides several academic implications as well as important practical implications for health care providers and patients. First, this study is the first attempt to examine the depression screening performance of mobile mental-health trackers, which collect daily mental-health ratings from patients. Our results show that the depression screening performance of mobile mental-health trackers is comparable to the traditional method, administration of a PHQ-9 test, in the clinical setting.

There may be a concern about collecting data with a small number of questions related to depressive tendencies. However, based on our findings, we argue that the portability of mobile phones, which enable patients to report their mental-health ratings on a daily basis, compensates for this disadvantage. The memory recall issue is particularly critical for cancer patients because their mental status is often unstable due to the side effects of cancer treatment [48,49]. A shorter recall period may reduce the potential for measurement errors and offset the limitation of a shorter survey. Another concern may involve the inconvenience for patients to report mental-health ratings every day. However, the use of a facial emoticon scale should reduce this inconvenience. Prior studies in psychology suggest that using a face emoticon scale demands less cognitive effort and is less of a burden to patients in interpreting questionnaire items [50,51]. Also, a face emoticon scale can actually make participation in the survey more enjoyable [52]. Therefore, the use of a face emoticon scale adapted to the small phone screen may facilitate user participation, potentially making the data more useful.

Second, this study provides empirical evidence that patient adherence to self-reporting via mobile mental-health trackers has a positive effect on the depression screening accuracy. For clinicians, it might be inefficient to analyze a large number of PROs obtained from various sources such as mobile or wearable devices because these data are susceptible to common instrumentation pitfalls such as noise, bias, and variability [21]. To design a systematic approach to distinguish meaningful PROs from noises, we employed the concept of adherence because it is known that adherent patients tend to make more efforts to successfully comply with suggested treatment guidelines [22]. The PROs reported by patients with higher adherence tend to be of high quality and quantity, and our results show that the accuracy of depression screening is higher for those patients [23].

Third, we provide a new perspective on measuring adherence to self-reporting as a multidimensional construct, consisting of activeness, timeliness, and persistence. Prior empirical studies of adherence to mobile PROs have tended to focus on the total number of PROs only (activeness) without considering that the overall adherence level can decrease over time [10,40]. By incorporating the degree of patient autonomy in timely reporting (timeliness) over the entire treatment period (persistence), this new measurement allows us to capture the time effects over both short- and long-term horizons. Our empirical analysis shows that the difference in screening accuracy between high- and low-adherence groups is clearer when the groups are classified based on three dimensions (activeness, timeliness, persistence) than when they are classified based on activeness only, supporting our argument that time dimensions are also important aspects of patient adherence.

Fourth, our results also have important implications for patients. Reporting daily mental-health ratings can be a significant burden for patients and can have an adverse effect on their mental status [53,54]. However, these burdens may be reduced if patients recognize the clinical benefits of reporting their outcomes (ie, PRO) [55]. Our results can help patients understand the positive effects of adherence and provide motivation for them to adhere to self-reporting and thereby improve the quality of treatment.

### Practical Implications

Our study provides clinicians with a practical guideline for transforming daily mental-health ratings reported via a mobile mental-health tracker to generate indicator variables for depression screening. As new technologies generate new types of data, doctors are challenged to deal with them. For instance, they may have questions such as “Should a variable be dichotomized? How should we determine the cut-off values?” and “How should we transform daily PROs into a biweekly format? Is a ratio approach better than a frequency approach?”.

These questions are not easy, and the answers vary depending on the situation. The average approach is easy for doctors to implement because it does not require clinicians to calculate the cut-off value [35]. Calculating the optimal cut-off value could be burdensome for them because cut-off values may be different according to demographics or the scale of the questionnaire [35]. Also, the optimal cut-off value cannot be calculated a priori without sufficient data. Thus, clinicians must wait for a certain amount of time until they obtain a sufficient amount of data to get the optimal cut-off value. This implies that the ratio and the frequency approaches cannot be used during early periods in which doctors are just starting to implement screening depression using daily mental-health ratings. Our empirical results show that the accuracy of depression screening by the average approach is clinically acceptable [39]. Thus, during the early period, using the average approach may be more appropriate.

As more data are accumulated, clinicians may choose the ratio or the frequency approach. In some cases, doctors may want to see a simple count of depressed days of a patient during a certain period in order to compare it with the results of traditional depression screening tests. In this case, although the scores of daily mental-health ratings as continuous variables, as well as the values obtained based on the average approach, may provide clinicians with much detailed information, doctors still need to determine whether the scores are high enough to consider a patient to be depressed [35]. Therefore, a systematic way to construct reasonable cut-offs is still needed, and we believe our proposed approaches (the ratio and the frequency approaches) and empirical results of their performance are valuable for clinicians.

It should be noted that the results of data processing depend on the nature of data, such as missing values and outliers, and each approach has its own limitations when dealing with these issues. For instance, the average approach is susceptible to outliers. The frequency approach considers depressed days only, ignoring the difference between normal days and days when ratings are not reported. The ratio approach considers the days when ratings are reported, ignoring the presence of omitted days. Thus, it is important to note that the relative superiority of data-processing approaches varies by situations. We recommend doctors choose an appropriate approach based on their clinical purposes.

### Limitations

Our study is a derivation study, and we still need future validation studies using different patient samples before this measure is more broadly adopted. First, we used three variables to evaluate mental health—sleep, mood, and anxiety—to gather information on patients’ mental status. Although we selected these three variables based on prior studies [24,25], there may be other important dimensions to assess daily mental status. However, to the best of our knowledge, there has been no study investigating which types of mental-health PROs should be considered for the mobile mental-health trackers. Therefore, a natural extension of our study would be to investigate the optimal choice of the mental dimensions to be used in mobile mental-health trackers. Second, we did not account for the methods of dealing with missing ratings. Further studies on this issue may be useful for improving the accuracy of depression screening in the mobile phone setting. For instance, future research may examine how the quantity of missing values affects the screening performance or how missing values can be effectively imputed to improve depression screening using ratings via mobile phone apps.

Third, our study was conducted in a breast cancer treatment setting in South Korea. Therefore, our results may not be generalizable to other types of mental illness or to patients with different diseases, especially patients with more severe malignancies, such as pancreatic and rectal cancers. Furthermore, South Korea is known for a high percentage of mobile phone users compared with other countries. Mobile app development technology and data management skills are considered to be of high quality. Therefore, the implications from our study may not be applicable in an environment where complementary infrastructures are not adequately supported. In this regard, our study warrants further research on the assessment of the use of mobile mental-health trackers in other settings.

### Conclusion

Self-reported daily mental-health ratings obtained via a mobile phone app can be used for screening for depression in breast cancer patients. Adherence to self-reporting can improve the efficacy of mobile phone‒based approaches for managing distress in this population.

## Appendix

Multimedia Appendix 1 Supplementary information for (1) number of days patients report logs in a 2-week period, (2) random-effects logistic panel regression models, and (3) k-means clustering algorithm.

## Abbreviations

AUC: area under the ROC curve
PHQ-9: Patient Health Questionnaire-9
PRO: patient reported outcome
ROC: receiver operating characteristic
