# Supplementary material for: Depression Screening Using Daily Mental-Health Ratings from a Smartphone Application for Breast Cancer Patients
Source: J Med Internet Res. 2016 Aug 4;18(8):e216. doi: 10.2196/jmir.5598 (PMC4990716; doi:10.2196/jmir.5598)
Supplement: Multimedia Appendix 1 [file jmir_v18i8e216_app1.pdf]

Appendix 1:
The Number of Days When Patients Report Logs in A Two-Week Period

The number of days patients report logs	Frequency	%	Cumulative %
1	10	2.01	2.01
2	8	1.61	3.62
3	12	2.41	6.04
4	14	2.82	8.85
5	15	3.02	11.87
6	17	3.42	15.29
7	25	5.03	20.32
8	17	3.42	23.74
9	20	4.02	27.77
10	33	6.64	34.41
11	43	8.65	43.06
12	57	11.47	54.53
13	90	18.11	72.64
14	136	27.36	100
Total	497	100	

Frequency: The number of cases when a patient reports logs on the number of days of each interval during a two-week period.
%: The percentage of cases in each interval.
Cumulative %: The sum of all the percentage values up to the interval.

Random-Effects Logistic Panel Regression Models

A logistic regression is a regression model in which the dependent variable is binary. The model is used to estimate the probability of the dependent variable being one based on explanatory variables.

A logistic panel regression is used when it deals with cross-section, time-series panel data collected from the same individuals over time. A panel analysis should account for individual heterogeneity because individual characteristics may affect the estimation result.

One of the approaches to deal with individual heterogeneity is to employ a fixed-effect model. A fixed-effect model deals with this issue by estimating all individual intercept terms β_{1i} for each individual *i*. Thus, it is inefficient to estimate all intercept terms when there are relatively many individuals. Also, observations are excluded if there is no variation in variables (i.e., time-invariant variables, such as gender) because the model estimates coefficients by using only time-variant variables within an individual.

The other approach is to use a random effect model. A random effect model assumes a random sampling process and considers β_{1i} to include two components: β_1 and v_i

($\beta_{1i} = \beta_1 + v_i$). Here, β_1 denotes the average expected odds ratio when other covariates take zero values, and v_i represents an individual's random error (individual heterogeneity) deviated from the population. A random effect model does not require that all individual intercept terms be estimated. Also, time-invariant covariates can be estimated. A random effect logistic regression is formally specified as below:

$$ln\left(\frac{P(y_{it} = 1|x_{it}, v_i, e_{it})}{P(y_{it} = 0|x_{it}, v_i, e_{it})}\right) = \beta_1 + \sum_{k=1}^{K} \beta_k\, x_{kit} + v_i + e_{it}$$

In our model, y_{it} indicates an individual *i'* depression state (i.e., 1 = depressed and 0 = normal) at time *t,* and the term on the left-hand side is the log odds ratio that a patient is depressed (y_{it}=1).

On the right-hand side, β_1 denotes an intercept term, which is the average expected odds ratio. x_{kit} is an observation of the k-th covariate (i.e., daily logs in our model—sleep satisfaction, mood, anxiety) for an individual *i* at time *t*, and β_k is a coefficient of the corresponding covariate. v_i represents unobserved individual heterogeneity, which is considered to be random. In other words, a random-effect model assumes zero mean, independency between each individual, and a constant variance of v_i ($\mathrm{E}(v_i) = 0, \mathrm{cov}(v_i, v_j) = 0, \mathrm{var}(v_i) = \sigma_v^2$). e_{it} indicates the idiosyncratic errors that change across *t* as well as across *i*.

Readers can run a random effect logistic regression model with most statistical software, such as Stata, R, and SAS. We ran our model with Stata by using command "xtlogit" with an option "re". "xtlogit" is a command to run a logistic regression model for panel data, and "re" is the option for a random effect model. For further information, we recommend four sources listed below.

1. Wooldridge, Jeffrey M. *Econometric Analysis of Cross Section and Panel Data*. Cambridge, MA: MIT Press, 2010.
2. Conaway, Mark R. "A Random Effects Model for Binary Data." *Biometrics* (1990): 317–28.
3. Stata manual "xtlogit". http://www.stata.com/manuals13/xtxtlogit.pdf

k-Means Clustering Algorithm

k-means clustering is a method to classify subjects into homogeneous subgroups where each observation belongs to the cluster with the nearest intracluster distance and with the largest intercluster distance. *k*-mean clustering partitions *n* observations into *k* heterogeneous subsets (clusters) to minimize the intracluster sum of squares.

$$\text{Min S} = \sum_{i=1}^{k} \sum_{x\, \in\, s_i} |x_n - \mu_i|^2$$

Here, x_n represents the nth dimensional vector and μ_i is the mean of points in S_i.

To help readers understand how the *k*-means clustering algorithm works, a simple graphic example is illustrated below with the assumption that each observation is a two-dimensional.

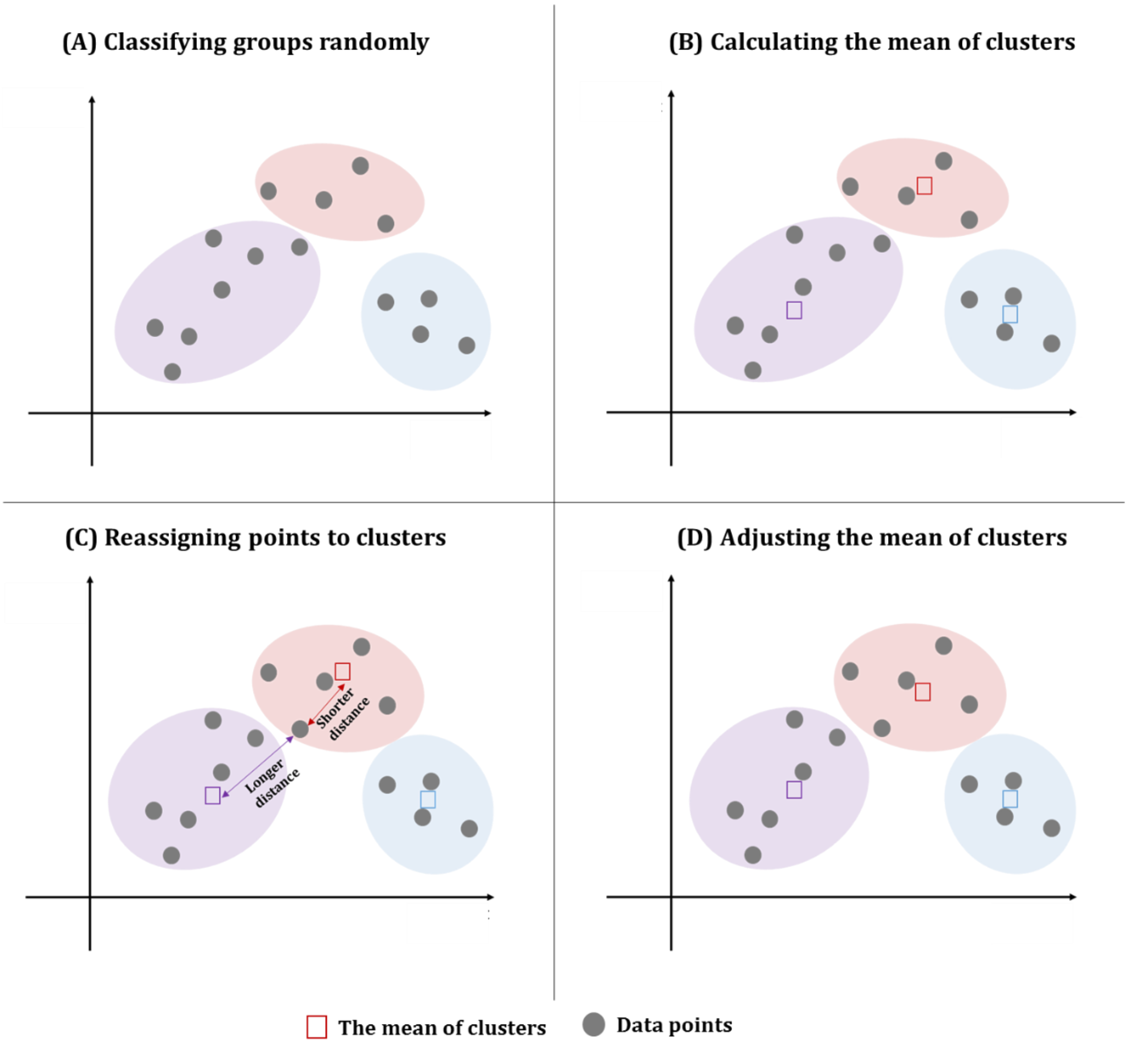

Figure 1. The procedure of *k*-means clustering

First, the *k*-means clustering algorithm classifies subgroups by randomly assigning x_n to k sets (A in figure 1). We chose three clusters in the example, but researchers can select any number (k) of clusters. Second, the algorithm calculates the mean of clusters as the centroids of the data points, the point that minimizes the within-cluster sum of squares (B in figure 1). Third, data points are reassigned to clusters of which the distance between the center of a cluster and a data point is the shortest (C in figure 1). Fourth, the mean of each cluster is recalculated (D in figure 1). Third and fourth steps (C and D in figure 1) are repeated until there is no further change.

Most statistical software, such as Stata, R, and SAS, provide *k*-means clustering algorithm modules. Stata users may run *k*-means clustering analysis by using the

command “cluster kmeans”. For further information about *k*-means clustering, we recommend the references listed below.

1. Hartigan JA, Wong MA. Algorithm AS 136: A K-Means Clustering Algorithm. *Journal of the Royal Statistical Society. Series C (Applied Statistics)* 1979;28(1):100–108.
2. Stata manual “cluster kmeans”. http://www.stata.com/manuals13/mvclusterkmeansandkmedians.pdf
